# Degradation of Phytosterols During Near-Ambient Drying of Rapeseeds in a Thick Immobile Layer

**DOI:** 10.1007/s11746-012-2065-3

**Published:** 2012-04-20

**Authors:** Marzena Gawrysiak-Witulska, Magdalena Rudzińska

**Affiliations:** 1 Faculty of Food Science and Nutrition, Poznan University of Life Sciences, Poznan, Poland; 2 Institute of Food Technology of Plant Origin, Poznan University of Life Sciences, Wojska Polskiego 31, 60-637 Poznan, Poland

**Keywords:** Rapeseed, Phytosterols, Near-ambient drying, Postharvest

## Abstract

The effect of the drying method applied and subsequent rapeseed storage on changes in phytosterols was determined. After harvest, rapeseeds were dried by the near-ambient method in a thick immobile layer of 2 m and using air heated to a temperature of 60, 80 and 100 °C. Analyses of phytosterol contents were performed immediately after drying and after 6 and 12 months of storage at a temperature of 10 ± 2 °C. Results showed a significant effect of drying conditions, cultivar-specific differences and storage time on the contents of phytosterols. Near-ambient drying of seeds resulted in a reduction in total sterol contents by 6–20 %, while for drying with hot air it was by 14–40 %. The level of sterols decreased by 13–18 % after a 1 year storage of seeds dried by the near-ambient methods. A reduction in 12–22 % in sterols for seeds dried by high temperature occurred after 1 year of storage.

## Introduction

Rapeseed is one of the most important oil crops, which seeds and fruits constitute raw material for the production of vegetable fats. In recent years rapeseed harvests have reached 58 million tonnes (http://www.faostat.fao.org). Postharvest moisture content of rapeseed may be as high as 18 %. However, this level is beyond the recommended storage moisture concentration. In Poland, it is recommended to dry seeds for long-term storage to a moisture content of 7 %; however, since 2011 in commercial turnover, transactions have been made on seeds with a moisture content of 9 %. The maximum moisture at which canola can be sold as straight grade (dry) is 10 % moisture [1–3]. A maximum moisture concentration at seed delivery of 8 % is recommended in Australia [4]. To achieve low moisture content the seeds must be dried.

According to Pathak et al. [5], the use of temperatures over 93 °C increases the content of free fatty acids in oil. Investigations conducted by Rudzińska et al. [6] showed that the higher the temperature of the drying agent, the greater the increments are in acid number and losses of phytosterols during hot-air drying. The application of high temperatures during drying of rapeseed may also significantly influence a reduction in their mechanical strength [7]. Since technological quality of rapeseed during drying and storage may deteriorate to a considerable degree, the drying method proposed with an increasing frequency for rapeseed is the energy-saving near-ambient drying in a thick immobile layer [8]. During near-ambient drying atmospheric air or air heated by several degrees Celsius is blown into a thick immobile seed layer (of several dozen to several meters in thickness). The air is heated when its drying potential is too low. The drying potential of air is analyzed automatically by the near-ambient drying controller. Seed drying occurs in a layer called the drying zone, which in the course of the process is shifted upwards from the bottom. For this reason in layers above the drying zone seeds have a moisture content similar to their initial moisture content [8, 9]. Since the process lasts typically from several to around a dozen days, there is a risk of quality deterioration and fungal growth [8, 10].

Rapeseed oil is a valuable source of biologically active compounds such as phytosterols, tocopherols and phenolic compounds. The concentration of phytosterols in seed oils does not exceed 1 % of total oil [11]. Also other vegetable oils, particularly maize oil, are very good source of phytosterols [12]. The profile of phytosterols is characteristic of the botanical species from which the oil was obtained and can be used to detect adulteration of commercial oil supplies [13]. An increased interest in products rich in phytosterols, expressed by consumers, enforces the need to minimize their losses during successive stages of postharvest processing of rapeseed. The drying method applied and the storage conditions may to a significant degree affect the degradation of biologically active compounds contained in rapeseed. Studies conducted by Gawrysiak-Witulska et al. [8] showed that during near-ambient drying losses of tocopherols amount to 6–11 %, while during high-temperature drying they amount to 4–8 %. There is a lack of data in available literature on the degradation of sterols during near-ambient drying of rapeseed. Thus the aim of these investigations was to determine the effect of the applied drying method and further storage of rapeseed on losses of phytosterols.

## Materials and Methods

### Materials

Analyses determining the effect of drying and storage conditions were conducted on three winter rapeseed cultivars: *Californium*, *Elektra* and *Livius.* Seeds were dried by either a near-ambient method in a 2-m immobile layer or by the high-temperature method.

Determinations of the phytosterol contents were performed directly after the completion of drying and after 6 and 12 months of seed storage at a temperature of 10 ± 2 °C. The bulk weight of stored rapeseeds was 1.5 kg. Seeds were stored in glass containers, in a room with minimal access to light.

The control comprised seeds immediately after harvest from the field.

### Near-Ambient Drying

Seeds of cv. *Californium* were dried by the near-ambient method as previously described [8, 14]. The initial seed moisture of 16.2 % was dried to 7 % after 136 h according to [8].

The *Elektra* and *Livius* seeds were dried under laboratory conditions, in a drier designed by Ryniecki et al. [15]. The near-ambient drying process was carried out following the method of [8] until the sample reached 7 % moisture content. Drying of seeds of *Livius* and *Electra* cv*.* was completed after 126 and 120 h, respectively.

Samples for analyses were collected after the completion of near-ambient drying from layers at the levels 0.1, 1.0, 1.5 and 2.0 m.

### High Temperature Drying

The seeds of the three cultivars were dried at 60, 80 and 100 °C in a thin layer of 0.005 m according to our previous method [8]. Samples were removed from the drier once the seed moisture content of 7 % was reached. Typical drying times of 12–15, 15–20, and 36–42 min were observed for temperatures of 100, 80 and 60 °C, respectively.

### Determination of Seed Moisture, Oil and Sterol Contents

The seed moisture was determined using a moisture balance following our previous method [8]. Oil extraction was completed using the Folch method [16] on 10 g ground rapeseed and 100 ml chloroform:methanol (2:1 v/v). The solvent was washed with 0.25 volumes of water, vortexed for several seconds and then centrifuged (2,000 rpm) to separate the two phases. The lower chloroform layer was collected and evaporated under vacuum using a Buchi R 215 rotoevaporator.

A saponification method of the AOCS [17] was used to determine the sterol composition. The GC and GC–MS methods of Ciftci et al. [18] were used to identify and quantify the individual sterols. These methods use a DB-35MS capillary column (25 m × 0.20 mm, 0.33 μm J&W Scientific, Folsom, CA) for a GC analysis and a DB-5 capillary column (50 m × 9 0.2 mm, 0.32 mm; J&W) for GC–MS. Other conditions used were as reported by Ciftci et al. [18].

### Statistical Analysis

Results are presented as means ± standard deviation from three replicates of each experiment. A *p* < 0.05 was applied to denote significant differences between mean values determined by the analysis of variance (ANOVA) with the assistance of Statistica 7.0 (StatSoft, Inc., Tulsa, OK) software.

## Results and Discussion

Five main phytosterols, i.e. brassicasterol, campesterol, stigmasterol, sitosterol and avenasterol, were identified in all samples. 4-monomethylsterols and 4,4′-dimethylsterols were not identified in lipid extracted from seeds.

**Fig. 1:**
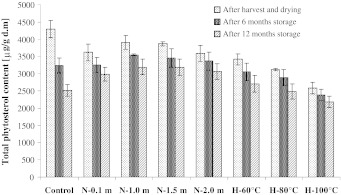
Changes of total phytosterol contents in analyzed rapeseed *Californium*. *N*, seeds dried by the near-ambient method (layer at 0.1…2 m), *H*, seeds dried by high temperature method (temperature from 60 to 100 °C)

**Fig. 2:**
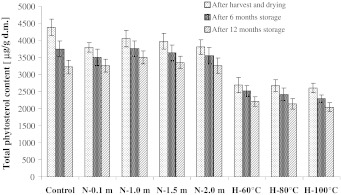
Changes of total phytosterol contents in analyzed rapeseed *Elektra*. *N*, seeds dried by the near-ambient method (layer at 0.1…2 m), *H*, seeds dried by high temperature method (temperature from 60 to 100 °C)

**Fig. 3:**
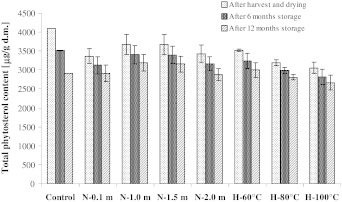
Changes of total phytosterol contents in analyzed rapeseed *Livius*. *N*, seeds dried by the near-ambient method (layer at 0.1…2 m), *H*, seeds dried by the high temperature method (temperature from 60 to 100 °C)

Changes in total sterol contents during drying and storage of rapeseeds are presented in Figs. 1, 2, 3, while those of individual sterols are given in Tables 1, 2, 3. In seeds harvested from the field, the total content of sterols was 4,298, 4,379 and 4,095 μg/g d.m for cv. *Californium*, *Elektra* and *Livius*, respectively. In all the tested cultivars, β-sitosterol was the dominant sterol, accounting for 46–54 % of the total sterol contents. For rapeseed cv. *Californium,* the content of β-sitosterol was 2,156 μg/g d.m. (50 %), for cv. *Elektra* 1,997 (46 %) μg/g d.m. and for cv. *Livius* it was 2,194 μg/g d.m. (54 %) seeds. The campesterol content in the fat extracted from seeds ranged from 1,246 μg/g d.m. in cv. *Livius* to 1,595 μg/g d.m. in cv. *Elektra* and it accounted for approx. 30–36% of the sterol fraction. Brassicasterol constituted approx. 13–16 % of the total sterols and its content ranged from 535 μg/g d.m. in cv. *Livius* to 686 μg/g d.m. in cv. *Elektra*. The other sterols were detected in much lower amounts. The content of avenasterol was 49–72 μg/g d.m., which constituted 1–2 % of the sterol fraction, while that of stigmasterol was 48–74 μg/g d.m. and did not exceed 2 % of their total contents. According to Rudzińska et al. [19], the total content of sterols in rapeseed is characterized by high variability depending on the cultivar and may range from 4,700 to 6,300 μg/g seeds. The differences in the contents of these compounds, apart from cultivar-specific traits, are also influenced by the moisture content of seeds, harvest and storage conditions [20]. According to Hamama et al. [21], rapeseed contains mainly sitosterol (45–60 %) and campesterol (25–39 %), as well as brassicasterol (5–13 %), avenasterol (3–7 %) and stigmasterol (<1 %). Similar data concerning the percentage shares of individual sterols were reported by Vlahakis and Hazebroek [20] and Rudzińska et al. [19].

**Table 1:** Changes of phytosterol contents in analyzed rapeseeds—*Californium*

Time	Control (μg/g d.m.)	Near-ambient drying				High temperature drying		
		0.1 m (μg/g d.m.)	1 m (μg/g d.m.)	1.5 m (μg/g d.m.)	2 m (μg/g d.m.)	60 °C (μg/g d.m.)	80 °C (μg/g d.m.)	100 °C (μg/g d.m.)
Brassicasterol								
Freshly harvested seeds	567 ± 57^c,3^	392 ± 28^ab,1^	417 ± 39^ab,1^	421 ± 35^ab,2^	421 ± 38^ab,1^	480 ± 42^bc,1^	410 ± 32^ab,2^	347 ± 26^a,1^
Stored for 6 months	381 ± 22^ab,2^	362 ± 27^ab,1^	399 ± 32^ab,1^	383 ± 31^ab,12^	413 ± 21^b,1^	434 ± 35^b,1^	389 ± 32^ab,12^	326 ± 24^a,1^
Stored for 12 months	273 ± 32^a,1^	343 ± 21^abc,1^	357 ± 32^bc,1^	341 ± 28^abc,1^	378 ± 32^c,1^	401 ± 38^c,1^	326 ± 27^abc,1^	293 ± 14^ab,1^
Campesterol								
Freshly harvested seeds	1,438 ± 73^d,3^	1,254 ± 84^cd,1^	1,361 ± 63^cd,2^	1,355 ± 63^cd,2^	1,197 ± 74^bc,1^	1,207 ± 92^bc,2^	1,040 ± 74^ab,1^	861 ± 63^a,1^
Stored for 6 months	1,121 ± 84^bc,2^	1,179 ± 89^bc,1^	1,271 ± 95^c,12^	1,231 ± 109^bc,12^	1,061 ± 105^abc,1^	1,092 ± 105^bc,12^	994 ± 87^ab,1^	819 ± 72^a,1^
Stored for 12 months	833 ± 53^ab,1^	1,071 ± 74^cd,1^	1,155 ± 84^d,1^	1,126 ± 116 ^cd,1^	1,007 ± 84^bcd,1^	966 ± 74^abcd,1^	917 ± 74^abc,1^	775 ± 63^a,1^
Stigmasterol								
Freshly harvested seeds	74 ± 5^d^	42 ± 4^bc^	51 ± 5^c^	49 ± 4^bc^	39 ± 3^b^	52 ± 4^c^	25 ± 2^a^	25 ± 3^a^
Stored for 6 months	nd	nd	nd	nd	nd	nd	nd	nd
Stored for 12 months	nd	nd	nd	nd	nd	nd	nd	nd
β-Sitosterol								
Freshly harvested seeds	2,156 ± 105^c,3^	1,890 ± 126^bc,2^	2,037 ± 95^c,2^	2,006 ± 156^c,1^	1,893 ± 116^bc,1^	1,638 ± 105^b,2^	1,607 ± 63^ab,2^	1,313 ± 74^a,2^
Stored for 6 months	1,687 ± 109^bc,2^	1,682 ± 97^bc,12^	1,848 ± 84^c,12^	1,809 ± 126^c,1^	1,853 ± 129^c,1^	1,491 ± 107^ab,12^	1,468 ± 116^ab,12^	1,211 ± 73^a,12^
Stored for 12 months	1,383 ± 76^bc,1^	1,547 ± 102^bcd,1^	1,659 ± 105^cd,1^	1,691 ± 85^d,1^	1,649 ± 105^cd,1^	1,313 ± 126^ab,1^	1,224 ± 97^ab,1^	1,092 ± 93^a,1^
Avenasterol								
Freshly harvested seeds	63 ± 6^b,3^	42 ± 3^a,2^	44 ± 3^a,2^	42 ± 3^a,3^	40 ± 2^a,2^	41 ± 2^a,2^	38 ± 3^a,3^	35 ± 4^a,2^
Stored for 6 months	48 ± 4^c,2^	32 ± 1^ab,1^	30 ± 2^a,1^	35 ± 2^ab,2^	39 ± 3^b,2^	32 ± 3^ab,1^	31 ± 2^a,2^	28 ± 2^a,2^
Stored for 12 months	25 ± 3^ab,1^	28 ± 3^b,1^	25 ± 2^ab,1^	26 ± 2^b,1^	31 ± 3^b,1^	26 ± 2^b,1^	18 ± 3^a,1^	18 ± 2^a,1^

Averages in rows followed by the same letter are not significantly different at the level α = 0.05

Averages in columns followed by the same number are not significantly different at the level α = 0.05

**Table 2:** Changes of phytosterol contents in analyzed rapeseeds—*Elektra*

Time	Control (μg/g d.m.)	Near-ambient drying				High temperature drying		
		0.1 m (μg/g d.m.)	1 m (μg/g d.m.)	1.5 m (μg/g d.m.)	2 m (μg/g d.m.)	60 °C (μg/g d.m.)	80 °C (μg/g d.m.)	100 °C (μg/g d.m.)
Brassicasterol								
Freshly harvested seeds	686 ± 52^c,2^	495 ± 43^b,2^	582 ± 42^bc,2^	572 ± 31^b,2^	520 ± 31^b,2^	382 ± 31^a,2^	368 ± 27^a,2^	354 ± 31^a,2^
Stored for 6 months	600 ± 36^d,12^	445 ± 35^b,12^	541 ± 28^cd,12^	520 ± 25^bcd,12^	486 ± 31^bc,12^	341 ± 28^a,12^	336 ± 25^a,12^	315 ± 21^a,12^
Stored for 12 months	504 ± 32^d,1^	396 ± 12^b,1^	481 ± 27^cd,1^	458 ± 22^bcd,1^	422 ± 35^bc,1^	296 ± 25^a,1^	302 ± 21^a,1^	274 ± 21^a,1^
Campesterol								
Freshly harvested seeds	1,595 ± 89^c,2^	1,394 ± 42^bc,2^	1,466 ± 94^bc,1^	1,414 ± 94^bc,2^	1,342 ± 94^b,1^	874 ± 73^a,1^	905 ± 52^a,1^	894 ± 52^a,2^
Stored for 6 months	1,391 ± 103^b,12^	1,315 ± 98^b,12^	1,363 ± 85^b,1^	1,302 ± 87^b,12^	1,243 ± 102^b,1^	840 ± 53^a,1^	830 ± 46^a,1^	809 ± 44^a,12^
Stored for 12 months	1,182 ± 79^b,1^	1,199 ± 68^b,1^	1,281 ± 82^b,1^	1,167 ± 75^b,1^	1,144 ± 84^b,1^	790 ± 58^a,1^	788 ± 53^a,1^	735 ± 32^a,1^
Stigmasterol								
Freshly harvested seeds	52 ± 4^d^	27 ± 2^ab^	34 ± 3^bc^	37 ± 3^c^	31 ± 2^abc^	24 ± 3^a^	28 ± 3^ab^	28 ± 3^ab^
Stored for 6 months	nd	nd	nd	nd	nd	nd	nd	nd
Stored for 12 months	nd	nd	nd	nd	nd	nd	nd	nd
β-Sitosterol								
Freshly harvested seeds	1,997 ± 104^b,2^	1,851 ± 42^b,2^	1,946 ± 106^b,1^	1,924 ± 104^b,2^	1,889 ± 88^b,1^	1,383 ± 114^a,1^	1,352 ± 93^a,1^	1,303 ± 60^a,2^
Stored for 6 months	1,736 ± 95^b,12^	1,721 ± 107^b,12^	1,833 ± 116^b,1^	1,791 ± 115^b,12^	1,801 ± 111^b,1^	1,313 ± 74^a,1^	1,229 ± 105^a,1^	1,145 ± 42^a,12^
Stored for 12 months	1,523 ± 84^b,1^	1,645 ± 109^b,1^	1,719 ± 72^b,1^	1,706 ± 85^b,1^	1,674 ± 106^b,1^	1,103 ± 53^a,1^	1,029 ± 84^a,1^	1,008 ± 74^a,1^
Avenasterol								
Freshly harvested seeds	49 ± 3^b,3^	28 ± 3^a,2^	26 ± 3^a,2^	25 ± 2^a,2^	25 ± 2^a,3^	26 ± 2^a,3^	27 ± 3^a,2^	26 ± 3^a,2^
Stored for 6 months	21 ± 0^a,2^	21 ± 1^a,1^	21 ± 0^a,12^	20 ± 2^a,12^	19 ± 2^a,2^	21 ± 0^a,2^	22 ± 2^a,1^	23 ± 2^a,12^
Stored for 12 months	11 ± 0^a,1^	18 ± 2^c,1^	18 ± 2^c,1^	16 ± 2^bc,1^	14 ± 1^ab,1^	17 ± 0^bc,1^	18 ± 0^c,1^	19 ± 2^c,1^

Averages in rows followed by the same letter are not significantly different at the level α = 0.05

Averages in columns followed by the same number are not significantly different at the level α = 0.05

**Table 3:** Changes of phytosterol contents in analyzed rapeseeds—*Livius*

Time	Control (μg/g d.m.)	Near-ambient drying				High temperature drying		
		0.1 m (μg/g d.m.)	1 m (μg/g d.m.)	1.5 m (μg/g d.m.)	2 m (μg/g d.m.)	60 °C (μg/g d.m.)	80 °C (μg/g d.m.)	100 °C (μg/g d.m.)
Brassicasterol								
Freshly harvested seeds	535 ± 48^b,2^	403 ± 26^a,1^	412 ± 35^a,1^	453 ± 31^ab,1^	398 ± 25^a,1^	421 ± 21^a,2^	418 ± 31^a,1^	412 ± 31^a,2^
Stored for 6 months	474 ± 43^b,12^	379 ± 28^a,1^	387 ± 32^a,1^	419 ± 32^ab,1^	371 ± 29^a,1^	390 ± 23^a,12^	391 ± 19^a,1^	377 ± 18^a,12^
Stored for 12 months	394 ± 35^a,1^	345 ± 25^a,1^	354 ± 28^a,1^	385 ± 31^a,1^	346 ± 27^a,1^	359 ± 21^a,1^	362 ± 17^a,1^	345 ± 17^a,1^
Campesterol								
Freshly harvested seeds	1,246 ± 72^b,2^	1,032 ± 68^a,2^	1,102 ± 82^ab,1^	1,109 ± 74^ab,1^	1,060 ± 82^ab,2^	1,071 ± 72^ab,2^	999 ± 41^a,2^	968 ± 52^a,1^
Stored for 6 months	1,092 ± 63^b,2^	949 ± 53^ab,12^	1,013 ± 79^ab,1^	1,042 ± 82^ab,1^	988 ± 36^ab,12^	991 ± 69^ab,12^	940 ± 57^ab,12^	893 ± 71^a,1^
Stored for 12 months	906 ± 52^a,1^	860 ± 48^a,1^	917 ± 72^a,1^	944 ± 75^a,1^	895 ± 33^a,1^	897 ± 62^a,1^	852 ± 52^a,1^	822 ± 65^a,1^
Stigmasterol								
Freshly harvested seeds	48 ± 4^c^	25 ± 3^ab^	26 ± 3^b^	27 ± 3^b^	26 ± 2^b^	30 ± 3^b^	27 ± 2^b^	18 ± 2^a^
Stored for 6 months	nd	nd	nd	nd	nd	nd	nd	nd
Stored for 12 months	nd	nd	nd	nd	nd	nd	nd	nd
β-Sitosterol								
Freshly harvested seeds	2,194 ± 134^b,3^	1,870 ± 94^ab,1^	2,091 ± 144^b,1^	2,042 ± 155^b,1^	1,896 ± 124^ab,2^	1,947 ± 124^ab,1^	1,700 ± 82^a,2^	1,627 ± 62^a,1^
Stored for 6 months	1,887 ± 115^bc,2^	1,758 ± 147^abc,1^	1,965 ± 126^c,1^	1,899 ± 112^bc,1^	1,771 ± 116^abc,1^	1,807 ± 105^abc,1^	1,618 ± 36^ab,12^	1,521 ± 114^a,1^
Stored for 12 months	1,566 ± 96^ab,1^	1,677 ± 140^abc,1^	1,886 ± 121^c,1^	1,791 ± 106^bc,1^	1,605 ± 105^abc,1^	1,699 ± 102^abc,1^	1,554 ± 34^ab,1^	1,475 ± 111^a,1^
Avenasterol								
Freshly harvested seeds	72 ± 5^c,2^	41 ± 3^b,2^	45 ± 3^b,2^	46 ± 3^b,1^	42 ± 1^b,1^	50 ± 4^b,1^	49 ± 3^b,2^	31 ± 3^a,2^
Stored for 6 months	61 ± 4^c,1^	37 ± 2^b,12^	42 ± 2^b,12^	42 ± 3^b,1^	38 ± 3^b,1^	45 ± 4^b,1^	42 ± 2^b,1^	25 ± 2^a,1^
Stored for 12 months	50 ± 3^d,1^	33 ± 3^b,1^	39 ± 2^bc,1^	39 ± 2^bc,1^	35 ± 3^bc,1^	41 ± 4^c,1^	38 ± 2^bc,1^	23 ± 2^a,1^

Averages in rows followed by the same letter are not significantly different at the level α = 0.05

Averages in columns followed by the same number are not significantly different at the level α = 0.05

After the completion of rapeseed near-ambient drying, the total content of sterols in samples collected for analyses decreased in comparison to the control samples by 7–20 %. In fat extracted from seeds of cv. *Californium*, the total content of phytosterols ranged from 3,590 to 3,909 μg/g d.m. depending on the dried layer. For cv. *Livius* and *Electra* their contents amounted to 3,371–3,677 μg/g d.m. and 3,795–4,055 μg/g d.m., respectively. The greatest losses of phytosterols during near-ambient drying were recorded in seeds dried at the level of 0.1 and 2 m, i.e. for cv. *Californium* (14–16 %), *Livius* (16–18 %) and *Elektra* (13 %). In the layers of seeds dried at the levels of 1 and 1.5 m, losses of total sterols were lower and did not exceed 10 %. However, statistical analysis showed that the content of phytosterols did not differ significantly (*p* > 0.05) in seeds dried at individual levels of the near-ambient drier.

In the course of near-ambient drying the greatest degradation rates were observed for stigmasterol and avenasterol. Their losses during drying in the layers at 0.1 and 2 m amounted to 40–48 % (stigmasterol) and 34–49 % (avenasterol). Losses of brassicasterol during near-ambient drying were higher than those of sitosterol and campesterol. In the layers at the level of 0.1 and 2 m they amounted to 24–31 %, while in the layer at the level of 1 and 1.5 m it was 15–26 %. Stigmasterol, avenasterol and brassicasterol have two double bonds in their molecules. This may have a significant effect on a more rapid degradation of these compounds during drying of rapeseed. Degradation of campesterol and sitosterol was lower. The content of campesterol during near-ambient drying of seeds was reduced in the layers at 0.1 and 2 m by 13–17 %, while in the other layers it was by 5–12 %. Losses of sitosterol in the layers at the level of 0.1 and 2 m in seeds of cv. *Californium* amounted to 12 %, in cv. *Livius* a loss of 14–15 % was observed, while in cv. *Elektra* it was 5–7 %. The loss of sitosterol in seeds dried by the near-ambient method in the layers at the level of 1 and 1.5 m, for all the tested cultivars, did not exceed 7 %. The analysis of losses for individual sterols also showed greatest losses of these compounds at the level of 0.1 and 2 m. However, these values varied between individual sterols.

Near-ambient drying lasts from several to around a dozen days. Recorded results made it possible to present in the graphic form the changes in seed moisture contents at selected levels of the drier [8]. During the experiment the drying front, characteristic of near-ambient drying, was observed to move upwards from the bottom. This occurred because air flowing through the bottom layers of rapeseed collected moisture from the seeds and when reaching the upper layer it no longer had the drying potential. Only after drying the bottom layers the flowing air started to absorb moisture from seeds found in the upper layers. A reduction in moisture content in seeds from individual layers was accompanied by an increase in temperature. In the conducted evaluations seeds were dried in a layer of approx. 2 m in thickness (i.e. the layer used during rapeseed drying on farms). Seeds of cv. *Livius* and *Elektra* at the level of 0.1 m reached the desired moisture content of 7 % after 6 h, while at the level of 1 m it was after 40–48 h, and at 1.5 m after 64–78 h. At the level of 2 m, seeds required 120–126 h from the beginning of the process to reach 7 % moisture. The longest drying process was carried out for seeds of cv. *Californium.* Their initial moisture content of 16.2 % extended the duration of the experiment to 163 h. Drying curves for seeds of rape cv. *Californium* were similar to those for seeds of cv. *Livius* and *Elektra* due to identical layer thicknesses (2 m) and a similar character of changes in drying air parameters in the course of the experiments. Thus seeds dried in a BIN silo and in the laboratory drier were equally exposed to the risk of oxidation changes.

From the time when the drying front reached the 0.1 m layer to the completion of the process (more than 110 h) the temperature of the seed mass was identical to that of the drying air (28–35 °C) [8]. Such a situation probably caused an increased degradation of sterols in the layers at the 0.1-m level. An additional adverse factor during near-ambient drying is connected with the possible overdrying of seeds in the bottom layers, if during the process atmospheric air has a very low relative humidity and a high temperature. In the discussed experiments, due to the atmospheric conditions found, seeds in the lower layers were overdried, as after the completion of drying their moisture content was 5.5 % [8]. Overdried seeds have a considerably reduced mechanical strength, which increases their susceptibility to damage during transport [22].

An increased risk of adverse biochemical changes during near-ambient drying is also observed in the upper layers. The higher the rapeseed layer, the longer the duration of the process and the longer the seeds have a high moisture content. The analysis of total sterol contents showed that losses of these compounds in the layer at the level of 2 m were comparable to those at the level of 0.1 m. In studies concerning the degradation of tocopherols during near-ambient drying different dependencies were obtained. The higher a given layer was located, the greater were the losses of these compounds [8].

Drying of rapeseed using hot air also caused degradation of these compounds. For cv. *Californium* and *Livius* these losses were proportional to the applied drying temperature and amounted to 21–40 % and 14–25 %, respectively (Figs. 1, 3). Total sterol losses during high-temperature drying of seeds of the *Elektra* cv. were similar (Fig. 2) and independent of the applied drying temperature. In all the samples analyzed during high-temperature drying, losses of total sterols were greater than during near-ambient drying. In seeds of cv. *Californium* and *Livius* during high-temperature drying (similar to during near-ambient drying) the highest degradation rates were observed for stigmasterol (37–66 %) and avenasterol (37–51 %). In seeds of cv. *Elektra*, dried using the high-temperature method, losses of stigmasterol, avenasterol, brassicasterol and campesterol were similar and amounted to 43–54 %. Losses of sitosterol in those seeds were lower (31–35 %).

The contents of phytosterols decreased in rapeseed dried using both methods and stored for 1 year. In seeds dried using the near-ambient method after 12 months of storage 13–18 % phytosterols were degraded, while in seeds dried using the high-temperature method it was 12–22 %. The greatest degradation rate was found for stigmasterol. It could no longer be detected after as little as 6 months of storage. According to Rudzińska et al. [6], losses of total sterols during 12 months of storage of dried seeds may reach 13 %, with stigmasterol and avenasterol undergoing the fastest degradation. Both storage time and temperature have a significant effect on the degradation of sterols [23]. Oxidized derivatives of sterols may be formed during degradation, which are considered to have a negative effect on the human organism [11].

## Conclusions

Rapeseed contains compounds of great value from the point of view of nutrition, i.e. phytosterols. In order to produce high quality oil from rapeseed it is necessary to minimize losses of these compounds during successive stages of postharvest processing. Drying costs have a significant share in the total outlays on rapeseed production, thus near-ambient drying in a thick immobile bed is being recommended with increasing frequency. Conducted analyses showed that, during near-ambient drying of rapeseed, losses of phytosterols may be smaller or similar to losses of these compounds occurring during high temperature drying of rapeseed. This makes it possible to recommend the near-ambient drying method as being advantageous in the process of postharvest processing.

## References

[1] Canadian Grain Commission (1994). Official grain grading guide.

[2] Canola Council of Canada (1981). Canola storage. Canola Council of Canada, Winnipeg, Canola Farming Fact Sheet 4. p 2

[3] Cass, J (1991) Canola storage: tips to help you through harvest. Intermountain Canola Newsletter, Fall 1991. Idaho Falls, Idaho. pp 3–4

[4] Mailer RJ, Colton RT, O’Bree BL (1998) Quality of Australian Canola. Canola Association of Australia. ISSN 1322-9397

[5] Pathak PK, Agrawal YC, Singh BPN (1991). Effect of elevated drying temperature on rapeseed oil quality. J Am Oil Chem Soc.

[6] Rudzińska M, Jeleń H, Nogala-Kałucka M, Gawrysiak-Witulska M (2006). The influence of storage time and drying temperature on sterols content in seeds of rapeseed. Oilseed Crops.

[7] Tys J, Sobczuk H, Rybacki R (2002). Influence of drying temperature on mechanical properties of seeds of oilseed rape. Oilseed Crops.

[8] Gawrysiak-Witulska M, Siger A, Nogala-Kalucka M (2009). Degradation of tocopherols during near-ambient rapeseed drying. J Food Lipid.

[9] Nellist ME (1998). Bulk storage drying in theory and practice. J Royal Agric Soc Engl.

[10] Ryniecki A (2005) Drying and Cooling Grain in Bulk-Handbook (Part 1). Mr Info, Poznań, Poland & KBN Handelsselskab v/Karlo B. Nielsen, Esbjerg, Denmark

[11] Ryan E, McCarthy FO, Maguire AR, O’Brien NM (2009). Phytosterol oxidation products: their formation, occurrence and biological effect. Food Rev Int.

[12] Piironen V, Toino J, Lampi AM (2000). Natural sources of dietary plant sterol. J Food Compos Analysis.

[13] Bortolomeazzi R, De Zan M, Pizzale L, Conte LS (1999). Mass spectrometry characterization of the 5α-, 7α-, and 7β-hydroxy derivatives of β-sitosterol, campesterol, stigmasterol, and brassicasterol. J Agric Food Chem.

[14] Gawrysiak-Witulska M, Wawrzyniak J, Ryniecki A, Perkowski J (2008). Relationship of ergosterol content and fungal contamination and assessment of technological quality of malting barley preserved in a metal silo using the near-ambient method. J Stored Prod Res.

[15] Ryniecki A, Gawrysiak-Witulska M, Wawrzyniak J (2007). Correlation for the automatic identification of drying endpoint in near-ambient dryers: application to malting barley. Biosystems Eng.

[16] Folch J, Lees M, Stanley GHS (1957). A simple method for the isolation and purification of total lipids from animal tissue. J Biol Chem.

[17] AOCS Official Method Ch 6-91 (1997) Determination of the composition of the sterol fraction of animal and vegetable oils and fats by TLC and capillary GLC

[18] Ciftci ON, Przybylski R, Rudzińska M, Acharya S (2011). Characterization of fenugreek (*Trigonella foenum*-*graecum*) seed lipids. J Am Oil Chem Soc.

[19] Rudzińska M, Muśnicki C, Wąsowicz E (2003). Phytosterols and their oxidized derivatives in seeds of winter oilseed rape. Oilseed Crops.

[20] Vlahakis C, Hazebroek J (2000). Phytosterol accumulation in canola, sunflower, and soybean oils: effects of genetics, planting location, and temperature. J Am Oil Chem Soc.

[21] Hamama AA, Bhardwaj HL, Starner DE (2003). Genotype and growing location effects on phytosterols in canola oil. J Am Oil Chem Soc.

[22] Tys J, Rybacki R (2001). Rzepak—Jakość nasion. Procesy zbioru, suszenia i przechowywania (Rapeseed—Seed quality. Harvesting, drying and storage processes). Acta Agrophysica.

[23] Rudzińska M, Przybylski R, Wąsowicz E (2009). Products formed during thermo-oxidative degradation of phytosterols. J Am Oil Chem Soc.

